# Psychological well-being of infertile women and its relationship with demographic factors and fertility history: a cross-sectional study

**DOI:** 10.1186/s12905-020-01167-3

**Published:** 2021-01-12

**Authors:** Farnaz Sohbati, Seyedeh Batool Hasanpoor-Azghady, Mina Jafarabadi, Leila Amiri-Farahani, Marzieh Mohebbi

**Affiliations:** 1 Department of Midwifery and Reproductive, Nursing Care Research Center (NCRC), School of Nursing and Midwifery, Iran University of Medical Sciences, Rashid Yasemi St., Valiasr St., Tehran, 1996713883 Iran; 2 Department of Obstetrics and Gynecology, Reproductive Health Research Center, Tehran University of Medical Sciences, Tehran, Iran; 3 Vali-Asr Reproductive Health Research Center, Imam Khomeini Hospital, Tehran University of Medical Sciences, Tehran, Iran

**Keywords:** Psychological well-being, Infertile women, Demographic factors, Fertility factors

## Abstract

**Background:**

Infertility leads to a wide range of psychological injuries that may reduce psychological well-being. This study aimed to determine the psychological well-being of infertile women and its relation with demographic factors and fertility history.

**Methods:**

This cross-sectional study was conducted on 300 infertile women referred to three infertility centres, Tehran, Iran. The sampling was continuous. We collected data from a self-generated demographic and fertility questionnaire and Ryff's Psychological Well-being Scale (PWB). Data analysis was done by independent t-test, one way ANOVA. The significance level was set at *P* < 0.05.

**Results:**

The results showed that there was no significant relationship between demographic variables including age, occupation of each couple, spousal’s education, economic status and place of residence with PWB, but the mean score of PWB was significantly different in women's educational levels (*P* = 0.03). There was also a significant difference between the mean score of PWB among different groups in the duration of marriage (*P* = 0.01). Fertility characteristics variables include the duration of infertility, duration of treatment of infertility, and current treatment were not the relation with PWB. However, the mean score of PWB in the number of IVF (*P* = 0.003) and the failed IVF pregnancies (*P* = 0.01) had a significant statistical difference.

**Conclusion:**

The results showed that PWB related to several variables. Paying attention to these variables can help in the preparation and development of counseling or educational programs.

## Background

Parenting is one of factor giving human beings a sense of wholeness [1] and pregnancy and childbearing are among the principal changes in women’s lives. For this reason, infertility can be a barrier to self-acceptance in some women [2]. Almost 10% of the world’s population is suffering from infertility. The prevalence of infertility in Iran is 20.2%; furthermore, this value in urban and rural areas is 19.9% and 22%, respectively [3].

Infertility and its treatment have a stressful nature. Stress resulting from infertility sometimes affects positive personal and social relationships, self-acceptance and purpose in life that decrease PWB [4, 5]. PWB requires a perception of the existential challenges of life. This approach examines the growth and development of the individual in the face of the existential challenges of life [6]. Reef defines psychological well-being as a positive perception of life challenges and efforts to realize true potential. He and his colleagues identified six factors of self-acceptance, purpose in life, personal growth, positive relations with others, environmental mastery and autonomy as components of PWB [7].

The results of some studies showed that infertile women had lower mean scores in the components of positive relations with others, purpose in life, self-acceptance, environmental mastery and personal growth compared to fertile women [4, 8]. For this reason, infertility is a risk to the PWB of infertile women [4, 9]. Infertility reduces the sense of autonomy because it exposes women to social pressures [10], somehow that some infertile women evaluate themselves through the lens of social standards. Fertile women feel more confident in the face of social pressures, which is reinforced by the tasks ahead of them. Many child-related duties force them to engage in creative activities. They also feel free to choose better options for themselves and their children, while infertile women sometimes succumb to complex environments [9].

For women, pregnancy is often a means to self-actualization [4]. Therefore, women who lack fertility may feel powerless, worthless, and inadequate, which leads to a decrease in their level of self-acceptance [11]. If motherhood is a desirable and valuable social position, infertility can be as an obstacle to achieving an important life goal for women [12]. Some infertile women report that infertility is the main issue in their lives so that they cannot move forward in life [13]. In Iranian society, many women learn many things after becoming mothers. The ability to do these things leads to self-satisfaction due to changes in the level of their abilities. Becoming a mother, on the other hand, gives structure to life and provides clear roles and social functions for women with greater control over various aspects of life [4, 11]. However, infertile women do not see more changes in their lives after marriage. Sadik believes this subject causes infertile women to allow life to go on in the same way instead of emphasizing their personal growth [9]. The stresses of infertility, as well as engaging in medical treatment and its longevity, cause psychological and social reactions in women that can take up their energy and time and limit their interests, efforts and opportunities to grow [2]. This issue can also affect the quality of life and attitude towards the social environment and reduce social and occupational abilities, and ultimately the sense of environmental mastery and purpose in life [14]. In the culture of our country, Iran, where families are large, one of the other challenges that infertile women face is the curiosity and pressures of those around them, which leads to little social communication and limited social network as a result of positive relations with others [8, 15]

Although many studies have shown the undesirable effects of infertility on mental health and PWB, few studies have shown a positive perception of the challenge of infertility. According to this view, women who have more control over their fertility problems reported higher PWB [16]. Also, the hope of successful treatment can give purpose to life and help a person adjust to her life [4]. The results of a qualitative study showed that a small number of infertile Iranian women reported that the infertility challenge increased their ability to have warm and intimate relationships with their husbands and families. Some others stated that infertility caused them to recognize more of themselves and choose goals in life according to their abilities. The process of achieving these goals has introduced them to new experiences and have led to their personal growth, especially in the spiritual dimension [2].

According to the literature review, it is necessary to take efficient measures to promote the PWB of infertile women. In this regard, it requires to identify the factors affecting PWB. Demographic data and infertility history are among the factors influencing this variable. Despite the importance of these factors, studies made little reference to it along with the main topic of their research [17]. PWB depends on various factors, such as age, socioeconomic status, urban or rural residence, duration of the marriage, duration of infertility, unsuccessful pregnancy, and kind of the treatment. Women with high employment and educational status face fewer difficulties in the family and society. However, women with low socioeconomic status face more problems and feel severely insecure about the future [2, 18]. The results of a study showed that old age reduces PWB by increasing the risks of treatment failure and reducing the chances of pregnancy [19]. Besides, older women with lower levels of education and unemployed have a lower quality of life than younger infertile women with higher levels of education and employment [20].

Type of treatment is another factor affecting PWB. In a study, Swedish infertile women reported unable to adapt themselves to childless condition, even three years after the end of an unsuccessful in vitro fertilization (IVF), and they could not get over their loss [21].

Although several studies have examined the effects of PWB on infertile women, men, and couples, few studies have investigated the relationship of demographic factors and history of infertility with PWB. Therefore, the consideration of these factors equips infertility treatment providers with a more comprehensive view to identify and help infertile couples by designing purposeful interventions. Accordingly, this study aimed to investigate the PWB of infertile women and its relationship with demographic factors and fertility history.

## Methods

This cross-sectional study enrolled 300 infertile women referring to the infertility centers of Imam Khomeini, Akbar Abadi, and Firoozgar Hospitals in Tehran, Iran. The sampling was carried out consecutively from January 2018 to September 2018. The inclusion criteria entailed:

(1) Iranian nationality, (2) reading and writing literacy to complete the questionnaires, (3) initial infertility with a female factor approved by the obstetrician, (4) a minimum of 1-year infertility treatment, (5) no adopted child, (6) lack of other medical illnesses unrelated to infertility, (7) absence of any mental illnesses requiring treatment based on the subjects' reports, (8) non-use of drugs, and (9) absence of any tension-generating events in the last 6 months.

The data collection tools included a self-generated demographic and fertility questionnaire and the 18-item version of Ryff's PWB Scale (short-form). This scale has six subscales. They are principal elements of psychological well-being. These subscales are autonomy, personal growth, environmental mastery, purpose in life, positive relations with others, and self-acceptance. Each subscale entails three items rated on a 6-point Likert scale (from strongly disagree = 1 to strongly agree = 6). Eight-item this scale are reverse scored. This scale has a score range of 18–108, with a higher score indicating better PWB [22]. In Iran, Khanjani et al. reported the internal consistency of this tool for autonomy, personal growth, environmental mastery, purpose in life, positive relations with others, and self-acceptance with Cronbach's alpha coefficients of 0.72, 0.73, 0.76, 0.52, 0.75, and 0.51 respectively [23]. We also used face and content validity for the validity of the demographic and fertility questionnaire.

The research project was confirmed by the Ethics Committee of Iran University of Medical Sciences, Tehran, Iran, with the ethics code of IR.IUMS.FMD.REC1396.9413373004. After obtaining a sampling license from Iran University of Medical Sciences, we started sampling at the infertility centers. Written consent obtained after an explanation of the purpose of the study and the confidentiality of the information. The data were analyzed in SPSS software (version 22) using independent t-test and one way ANOVA. The significance level for all tests was *P* < 0.05.

## Results

The mean and standard deviation of the age of the infertile women was 29.16 ± 5.81 years. Besides, the majority of the subjects (52%) had high school and relatively favorable economic status (65%). The mean and standard deviation of the total score of PWB reported in Table 1. Tables 2, 3, and 4 present more information about the demographic characteristics of the participants.

**Table 1:** Numerical indicators of PWB score based on different subscales in infertile women (n = 300)

PWB and its subscales	Mean	SD
Autonomy	10.91	1.85
Environment mastery	11.18	1.70
Self acceptance	11.18	1.70
Personal growth	10.72	1.79
Positive relations	10.13	2
Purpose in life	10.62	2.31
Total PWB score	64.75	5.31

**Table 2:** Frequency distribution of demographic characteristics and comparison of the mean score of PWB in terms of demographic characteristics (n = 300)

Characteristics	N (%)	Mean	SD	*P* value
Age (years)				
≤ 25	98 (32.7)	64.28	5.16	*P* = 0.7*
26–30	79 (26.3)	64.85	5.23	
31–35	79 (26.3)	65.22	5.7	
≥ 36	44 (14.7)	64.80	5.2	
Woman's education				
Elementary	14 (4.7)	65.86	6.53	*P* = 0.03*
Secondary school	98 (32.7)	63.78	4.76	
High school	156 (52)	64.84	5.49	
Academic	32 (10.7)	66.81	4.96	
Spousal's education				
Elementary	17 (5.7)	65.12	4.60	*P* = 0.5*
Secondary school	59 (19.7)	64.39	4.89	
High school	177 (59)	64.56	5.29	
Academic	47 (15.7)	65.79	6.12	
Place of residence				
City	281 (93.7)	64.81	5.39	*P* = 0.19**
Village	19 (6.3)	63.79	4.11	
Woman's occupation				
Housewife	211 (70.3)	64.93	5.31	*P* = 0.38**
Employed	89 (29.7)	64.31	5.33	
Spousal's occupation				
Employee	48 (16)	64.94	5.52	*P* = 0.11*
Free	119 (39.7)	65.50	5.49	
Manual worker	112 (37.3)	63.82	5.29	
Unemployed	21 (7)	65.00	3.05	
Marriage duration				
≤ 4.99	155 (51.7)	63.97	5.37	*P* = 0.01*
5–9.99	103 (34.3)	65.63	5.45	
10–14.99	28 (9.3)	64.32	4.35	
≤14.99	14(4.77)	63.97	5.37	
Economic status				
Favorable	53 (17.7)	65.89	6.11	*P* = 0.4*
Relatively favorable	195 (65)	64.52	5.07	
Undesirable	52 (17.3)	64.44	5.29	

*One way ANOVA

**Independent t-test

**Table 3:** Frequency distribution of fertility characteristics and comparison of the mean score of PWB in terms of fertility history (n = 300)

Characteristics	N (%)	Mean	SD	*P* value
Infertility duration				
≤ 4.99	230 (76.7)	64.45	5.39	*P* = 0.2*
5–9.99	54 (18)	65.72	5.03	
≥ 10	16 (5.3)	65.81	4.96	
Treatment Duration				
≤4.99	264 (88)	64.54	5.3	*P* = 0.9**
≥ 5	12 (36)	66.31	5.2	
Current treatment				
Drug	109 (36.3)	64.18	5.09	*P* = 0.17*
IUI	103 (34.3)	64.63	6.12	
IVF	88 (29.3)	65.59	4.45	

*One way ANOVA

**Independent t-test

Table 1 shows that the mean scores in the two subscales of environmental mastery and self-acceptance were higher than those of the other subscales. The mean score of total PWB calculated at 64.75, which is higher than the median score of the scale (63).

Table 2 shows a statistically significant difference in the mean scores of PWB in women of different educational levels (*P* = 0.03). In other words, women with academic education had more PWB than women with lower education. Moreover, there was a statistically significant difference in the mean scores of PWB in women with different marriage durations (*P* = 0.01).

Table 4 demonstrates a statistically significant difference in the mean scores of PWB regarding the number of IVF (*P* = 0.003) and the number of unsuccessful pregnancies with IVF (*P* = 0.01).

**Table 4:** Frequency distribution of fertility characteristics and comparison of mean scores of PWB in terms of fertility history in vitro fertilization treatment process (n = 88)

Characteristics	N (%)	Mean	SD	*P* value
Number of IVF***				
1	27 (30.6)	68	3.84	*P* = 0.003*
2	24 (27.2)	65.33	4.65	
3	18 (20.4)	63.5	5.17	
≥ 4	19 (21.5)	64.47	2.69	
Failed IVF pregnancies				
0	66 (75)	66.24	4.66	*P* = 0.01**
≥ 1	22 (25)	63.64	3.12	

*One way ANOVA

**Independent t-test

***Only 88 of all subjects had IVF

## Discussion

This study aimed to determine the PWB of infertile women and its relationship with demographic and fertility factors. According to the results, the mean score of PWB (64.75 ± 5.31; score range: 18–108) was higher than the median score of the scale (63). The highest and lowest mean scores of PWB were in the subscales of environmental mastery/self-acceptance (11.18 ± 1.70) and positive relations with others (10.13 ± 2), respectively.

Consistent with the results of the present study, in the research carried out by Rahmanifard et al. mean score of PWB was higher than the total median score of the scale [24]. However, the participants of the mentioned study scored better in each subscale, compared to those of the current study. Perhaps this difference is related to the level of education of the subjects of the two studies. In the mentioned study, 30% of the subjects had an academic education, while this value was 10.7% in the present study. In the same vein, the results of some other studies indicated that undereducated people have lower PWB [25, 26].

In another study conducted on infertile women in Tabriz, Iran, the total mean score of PWB was lower than the total median score of the scale; however, it was close to the value obtained in the present study (64.75) [4]. In our study, the mean score of the two subscales of environmental mastery and self-acceptance was higher than the median score of the subscale. However, in the mentioned study, the mean scores of the two subscales of purpose in life and autonomy were higher than the median score of the subscales. This difference may be due to the adoption of larger sample size in the current study. Moreover, the cultural context of Tabriz with mostly ethnic Turkish residents may have an impact.

In a study conducted by Jebraeli et al. the total mean score of PWB in infertile women in Meshkinshahr, Iran, was 45.04 ± 23.93 [27]. In another study conducted in Ardebil, Iran, the total mean score of PWB was 48.90 ± 1.44 [8]. The total mean score of PWB in the current study (64.75) was higher when compared with the results of the two mentioned studies. This difference may be because the participants of the present research referred to infertility centers from different parts of the country with various ethnicities. Nonetheless, the residents of Meshkinshahr and Ardebil have Turkish culture that puts the infertile women under social pressures due to the existing fertility demand, which in turn affects their PWB. On the other hand, the sample size of the current study is three times as many as those of the mentioned studies.

Our study indicated that the mean score of PWB was not statistically significant in terms of the different levels of variables, such as age, occupation of each couple, spousal’s education, and economic status. However, this difference was significant concerning women’s educational status (*P* = 0.03). In other words, women with academic education had more PWB than those with low education at other levels. In this regard, the results of a study conducted by Ma'roofizadeh et al. demonstrated that women with primary education had lower PWB as compared to those with academic education [26]. Besides, another research carried out in 34 European countries revealed that the prevalence of poor PWB is higher in undereducated infertile people [25].

Zurlo et al. emphasize the level of education as an important variable for two reasons. The first reason is related to a better perception of infertility and control of medical treatments, and the second reason is due to other happy aspects of life other than the mother that considered to be today [19]. On the other hand, the level of education of the infertile person is effective in how to cope with infertility. People with higher education, because of their ability to search for information sources, use more problem-solving coping strategies than people with less education [2]. Scientific documents also show that trying to find positive abilities and capabilities to cope with life's challenges leads to increased happiness in people. People who have experienced undesirable events in life, but focus on the positive aspects of life, report higher PWB [28, 29]. According to the results of the present study and related studies, it seems that facilities and conditions should be provided free of charge or at a low cost by the treatment team in infertility centers to replace the educational benefits for women with low levels of education. Such as being under the psychological support of psychologists, being in social networks where infertile counterparts are successful in coping with the infertility challenge or using other methods that help them pay attention to the components of PWB that they have a problem.

In the present study, the mean scores of PWB in different age groups of infertile women did not show a statistically significant difference. Results of a study carried out by Pazandeh et al. revealed that life satisfaction increases in infertile women with ageing. Furthermore, life satisfaction in the infertile women reported being at the highest level in the age range of 31–43 years [30]. In the present study, the mean score level of PWB in the age group of 31–35 years was higher than that of the other age groups; however, this difference was not statistically significant.

Regarding the economic status, the mean scores of the PWB of infertile women did not show a statistically significant difference in various economic status groups. In another research conducted by Dadkhahtehrani et al. there was no relationship between economic status and quality of life [31]. However, in the present study, people with a favorable economic status had more PWB. Nonetheless, regarding the frequency of these individuals (in total 17.7%), the mean scores of PWB of infertile women did not show a statistically significant difference in groups with different economic statuses.

Considering the place of residence, the mean score of PWB was lower in rural infertile women. Nonetheless, this difference was not statistically significant due to the small number of rural subjects (6.3%), compared to their urban peers. The results of some studies demonstrated that infertile women and couples living in rural areas reported lower PWB compared with the other subjects [2, 32].

In the present study, the mean score of PWB in different groups in terms of marriage duration was statistically significant. The increase of marriage duration led to the enhancement of the mean score of PWB. In this regard, the results of a study carried out by Pazandeh et al. revealed that the increase of the duration of marriage in the infertile group with the marriage age of 11–19 years improved the PWB status. In an attempt to justify this result, the research team of the mentioned study stated that infertile women deal with their inability to have a child over time and replace this desire with other alternatives [30]. Moreover, the results of another study conducted in Sweden demonstrated that 50% of infertile couples who underwent IVF and intracytoplasmic sperm injection (ICSI) treatments reported a significant improvement in their marital relationships during the infertility period [33]. According to the results of the present study and related studies, such as increasing the duration of marriage has replaced other purposes in life in life and better relationships between couples have been established, which has led to greater PWB. It is better to focus on these two components of PWB in counseling and psychological support. In this way, infertile women may be able to achieve these components in a shorter period.

In the present study, there was no statistically significant difference in the mean score of PWB in groups with different infertility and treatment durations. In another study conducted by Maroofizade et al. found no relationship between the duration of infertility and life satisfaction [34]. Nonetheless, another study conducted in Jordan reported a negative relationship between the duration of infertility and optimism [35]. On the contrary, the results of two other studies showed that a longer duration of infertility reduces anxiety and depression [36, 37]. The contradiction in the obtained results of these studies may be due to the assumption association between the history of infertility and dealing with this problem over time is linear and constant. However, experts emphasized that underlying factors (e.g., time, change, and personal resources) may form different patterns of coping with distress or non-linear patterns. Some studies have challenged the hypothesis regarding the linear effect of infertility history on cope and perception it [38].

In the present study, the mean PWB score in women who underwent IVF more than once was lower than the subjects who underwent IVF only once. Also, the mean PWB score in women who had more than once failed IVF pregnancies were lower than subjects not pregnancies by IVF. In line with the results of our study, Maroufizadeh et al. showed that PWB decreased with increasing frequency of treatment failure and in women who did not become pregnant with IVF was higher than infertile women who had failed IVF pregnancies once or twice [26]. The results of a qualitative study showed infertile women who receive IVF treatment because of receiving more hard treatment, failure treatment or failed IVF pregnancies feel out of control over their lives, which in turn reduces PWB [2].

## Research limitations

Because the data were based on self-reported answers by the subjects, the response to some items might have been influenced by cultural factors and society values.

## Conclusion

The obtained results demonstrated that psychological well-being was associated with demographic characteristics and infertility history, including educational levels, duration of marriage, number of treatment with IVF and number of failed IVF pregnancies. Therefore, the consideration of these variables can be helpful in the preparation and development of counseling or training programs.

## Abbreviations

PWB: Psychological well-being
IVF: In vitro fertilization
ICSI: Intracytoplasmic sperm injection

## Availability of data and materials

The datasets used and analyzed during the current study are available from the corresponding author on reasonable request.
